# *CNAdjust*: enhancing CNA calling accuracy through systematic baseline adjustment

**DOI:** 10.3389/fgene.2025.1674138

**Published:** 2025-09-26

**Authors:** Hangjia Zhao, Michael Baudis

**Affiliations:** ^1^ Department of Molecular Life Sciences, University of Zurich, Zurich, Switzerland; ^2^ Computational Oncogenomics Group, Swiss Institute of Bioinformatics, Zurich, Switzerland

**Keywords:** copy number alterations, baseline correction, bayesian framework, cancer genomics, nextflow workflow

## Abstract

Accurate determination of the genomic copy number baseline is crucial for identifying copy number alterations (CNAs) in cancer, yet it remains a significant challenge in tumors with complex karyotypes. To address this, we present *CNAdjust*, an integrated method to systematically detect and correct baseline inaccuracies in CNA data. *CNAdjust* employs a Bayesian framework that integrates cohort-specific CNA frequency priors with a data-driven plausibility score, ensuring that adjusted calls align with both biological cohort patterns and study-specific data. Performance validation using the TCGA pan-cancer dataset demonstrated improved alignment with absolute copy number estimates and enhanced CNA pattern interpretation. Furthermore, we revealed a strong correlation between chromosomal aneuploidy and baseline abnormalities, underscoring the prevalence of this issue in cancer genomics. By systematically improving the precision of CNA calls, *CNAdjust* serves as a critical tool for constructing harmonized reference datasets and advancing the progress of precision oncology. Its implementation as a standard, portable workflow enables the reproducible and scalable analysis of large, heterogeneous datasets, supporting large-scale genomic research. Source codes are available at: https://github.com/baudisgroup/CNAdjust.

## 1 Introduction

The term “Copy Number Alterations” (CNAs^1^) refers to changes in the copy number of genomic segments, representing deviations from the normal karyotype. CNAs are a hallmark of cancer cells and play a crucial role in cancer development (Sansregret et al., 2018). These variations are typically expressed as relative copy number ratios between test and reference samples. Normalizing this relative ratio is a critical step in data processing, as it corrects systematic technical variations and enables meaningful biological comparisons (Quackenbush, 2002).

For cancer cells with extensive copy number imbalances, traditional normalization methods, such as median or lowess normalization, often fail to maintain an accurate baseline. These shifts from the expected log ratio (logR) of 0 complicate relative CNA calling and lead to inaccuracies in downstream analyses. While density-based adjustments offer a solution (Staaf et al., 2007; Marzouka et al., 2016), they rely on the assumption that most genomic markers cluster near the true baseline—an assumption that fails in cancers with pervasive CNAs, such as adrenocortical tumors (Ronchi et al., 2013). Even more sophisticated single-sample calibration tools ultimately rely on similar assumptions, attempting to identify an anchor baseline from the sample’s internal data alone, implicitly relying on diploid, allelic-balanced regions or marker density dominance (Fu et al., 2015; Gao and Baudis, 2020). In highly aneuploid tumors, however, these assumptions are frequently violated, leading to systematic baseline errors.

The resulting unreliability of CNA detection tools is a well-documented problem. A comprehensive benchmarking study (Luo, 2019), for example, revealed that no single method performs consistently well across both near-diploid and highly aneuploid tumors, highlighting the critical need for self-adaptive strategies that can adjust to varying karyotypes. Moreover, the difficulty is not merely technical but also conceptual, especially in aneuploid samples. For instance, in a genome where half the regions have three copies and the other half have 2, setting the baseline at the average ploidy of 2.5 would label the entire genome as altered. While seemingly accurate in isolation, this becomes problematic if prior biological knowledge suggests a diploid background, as this incorrect baseline diminishes the significance of true alterations and obscures meaningful patterns.

To address these challenges, we introduce *CNAdjust*, a novel method designed to automatically detect and correct baseline abnormalities in tumor CNA profiles. *CNAdjust* employs a Bayesian framework that systematically evaluates potential baseline corrections. It integrates prior probabilities derived from cohort-level CNA patterns with a data-driven plausibility score calculated from the intra-study samples’ logR value distribution. This synergistic approach ensures that adjusted CNA calls are consistent with both broad biological context and study-specific evidence, leading to more accurate and reliable interpretations. Built on the Nextflow workflow management system, *CNAdjust* offers robust reproducibility, parallelism, and portability, making it an effective tool for calibrating large-scale and heterogeneous CNA datasets for oncogenomic studies.

## 2 Methods

### 2.1 Input data

**FIGURE 1:**
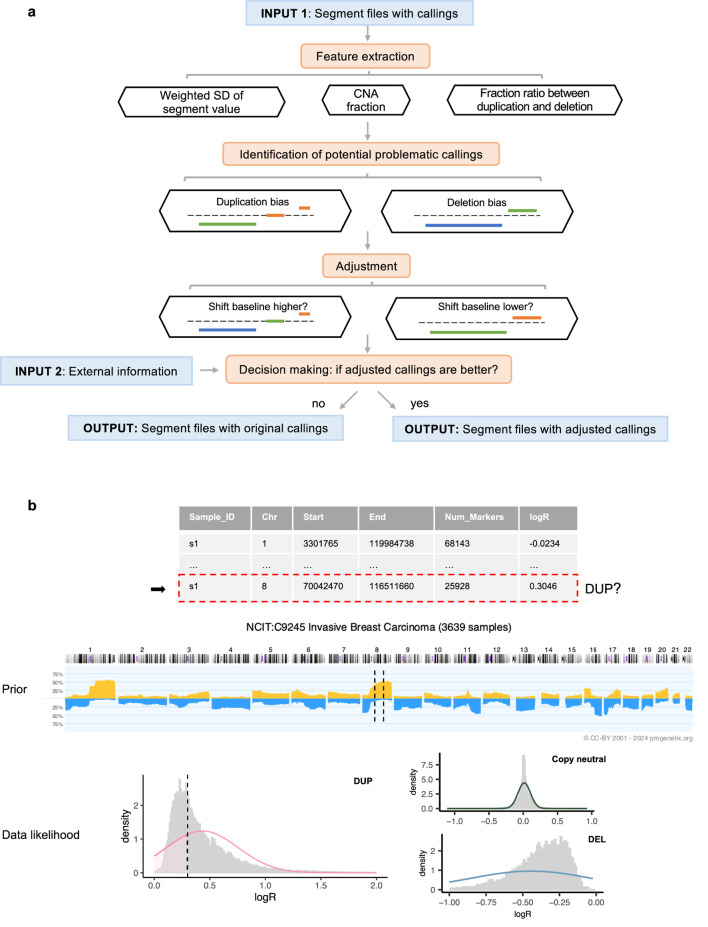
Overview of the workflow. **(a)** Execution steps, with segment colors indicating CNA states: blue for deletions, orange for duplications, and green for copy-neutral states. **(b)** Decision-making process for a segment identified as a duplication within a sample profile. In the middle prior pattern plot, dashed lines represent the regions spanned by the segment. In the bottom weighted logR distribution plot (by segment length), the dashed line indicates the logR value of the segment.

Our method is built on the observation that samples from the same cohort, such as those sharing a tumor (sub)type, typically display similar CNA patterns (Ni et al., 2013; Komori, 2022). Therefore, to refine the accuracy of CNA calls, the workflow requires external information, including cohort assignments for each sample and prior probabilities of gain and loss specific to each cohort (Figure 1a).

To demonstrate the applicability of our approach, we analyzed 9,846 masked copy number segment profiles from The Cancer Genome Atlas (TCGA), spanning 33 tumor types for which matched absolute copy number data was available. Two distinct sets of CNA calls are generated from this data set: the first derived from a straightforward cutoff approach using a threshold of 0.1, and the second using the same threshold but integrated with a density-based baseline correction using CopyNumber450kCancer (Marzouka et al., 2016). All samples from the same project were assigned to the same cohort group. To establish the prior probabilities essential for our model, we utilized CNA frequency data from Progenetix (Cai et al., 2012; Huang et al., 2021; Baudis Group, 2024), a comprehensive database that aggregates genomic mutation profiles from various sources, including Gene Expression Omnibus (GEO) (Barrett et al., 2012), cBioPortal (Cerami et al., 2012), and TCGA. The Progenetix database provides computed frequencies of CNAs for 1 MB genomic bins across samples from the same cohorts, thus providing the necessary prior information to reflect the CNA landscapes of specific populations. In our analysis, we used the National Cancer Institute Thesaurus (NCIt) terminology (National Cancer Institute, 2024) to match NCIt codes to different TCGA projects. Based on these NCIt codes, we retrieved the corresponding CNA frequency data from Progenetix, ensuring that the frequency values were derived from cohorts without any or containing only a small minority fraction of TCGA samples. Detailed information on the TCGA data and the corresponding NCIt codes can be found in the Supplementary Section 2 and Supplementary Table S2.

### 2.2 Workflow steps

The initial step is to individually analyze the segment profiles to identify those with potentially abnormal baselines. Aberrant baselines are indicated by pervasive CNA calls throughout the genome or extreme skewness in the CNA ratio. To detect these, this workflow extracts key features including the overall fraction of CNAs, the standard deviation of the segment value (logR) weighted by the number of markers, and the ratio of CNA fractions between duplications and deletions. The criteria for flagging problematic samples are based on an examination of 36 randomly selected GEO series that covered a variety of disease types and measurement platforms. For a detailed account of the test series and the derivation of default parameters, see the Supplementary Section 1. Our pipeline uses these default parameters, but they are designed to be user-adjustable.

For profiles identified with potential baseline issues, *CNAdjust* systematically corrects CNA calls by iteratively shifting the baseline upward or downward (Figure 1a). This core function is powered by our developed method, *labelSeg* (Zhao and Baudis, 2024), which uses unsupervised clustering to group segments based on their logR values. This clustering-based design makes the entire *CNAdjust* framework inherently adaptive to variations in tumor purity. Because purity primarily affects the amplitude of logR signals, the data-driven clustering identifies CNA states based on each sample’s own signal distribution, removing the need for an explicit purity prior. Rather than applying a single, fixed correction, *CNAdjust* conducts a systematic evaluation. The process begins by using the original calls to identify the initial baseline cluster. Depending on the detected bias, *CNAdjust* then directs *labelSeg* to select an adjacent cluster as a candidate for the new baseline, generating a new set of calls. This process can be repeated to test multiple candidate shifts.

Each set of candidate calls is then evaluated to determine which offers the greatest improvement. This decision-making process is automated by a Bayesian framework, as illustrated in Figure 1b and formalized in Equations 1,2, [Disp-formula e2]. Equation 1 shows the general principle of Maximum a Posteriori (MAP) estimation, where the most probable set of CNA states, ${\hat{\theta }}_{\mathrm{MAP}}$ is identified. To make this practical, we define an objective function, J(θ) , shown in Equation 2. This function assumes independence between the n genomic segments in a profile and is calculated by summing two components for each segment: a log-prior-probability and a log-plausibility-score. The prior probability, $p\left(\theta_{i}|r_{i},c\right)$ reflects the expected occurrence of a CNA state $\theta_{i}$ (duplication, deletion, or neutrality) in genomic region $r_{i}$ for a given cohort c . For demonstration purposes, we use CNA frequency data from Progenetix as our prior; however, the framework supports integrating any belief about CNA occurrence. The plausibility score, $S\left(d_{i}|\theta_{i},s\right)$ , substitutes for a formal likelihood and is derived from the tail probability of the observed logR value, $d_{i}$ , within the state-specific Gaussian distribution fitted on samples from the same study s . The final CNA calls are determined by finding the set of states θ that maximizes the objective function J(θ) , thus ensuring that the chosen calls reflect the most probable states given both the observed data and our prior knowledge. If no adjusted set is deemed superior to the original, the original calls are retained.

$${\hat{\theta }}_{\mathrm{MAP}}=\arg\underset{\theta }{\max}p\left(\theta |D\right)\propto \arg\underset{\theta }{\max}p\left(D|\theta \right)p\left(\theta \right)\propto \arg\underset{\theta }{\max}\log\left(p\left(D|\theta \right)p\left(\theta \right)\right)$$ (1)

$$J\left(\theta \right)=\overset{n}{\underset{i=1}{\sum }}\left[\log S\left(d_{i}|\theta_{i},s\right)+\log p\left(\theta_{i}|r_{i},c\right)\right]$$ (2)

## 3 Results

### 3.1 Performance validation

**FIGURE 2:**
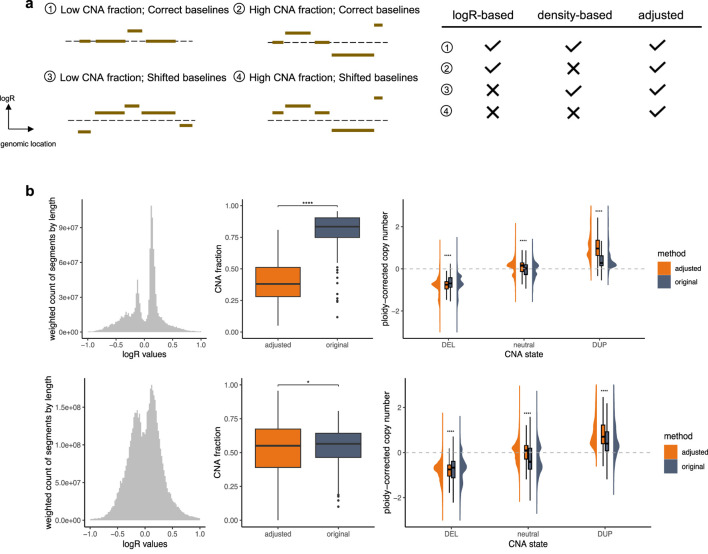
Performance Validation. **(a)** Different types of segment profiles. The dashed line represents the theoretical baseline. The accompanying table on the right summarizes the performance of various calling strategies across these profile types, emphasizing their challenges and the effectiveness of CNAdjust in addressing them. Checkmarks indicate reliable performance, while crosses denote problematic situations. **(b)** Samples with baseline issues. The top and bottom panels correspond to the logR-based and density-based calling groups, respectively. The left plot shows weighted histograms of logR values (weighted by segment length). The middle plot illustrates CNA fractions; statistical significance was determined using a paired t-test with Benjamini–Hochberg correction. The right plot depicts ploidy-corrected copy changes across different calling states; statistical significance was determined using a t-test with Benjamini–Hochberg correction.

To validate the performance of our approach and emphasize its utility, we analyzed TCGA pan-cancer segment data using two distinct methods for generating input callings: logR-based calling, which applies a direct 0.1 cutoff value and assumes the theoretical baseline (0) as correct, and density-based calling, which employs a density-based baseline correction using CopyNumber450kCancer (Marzouka et al., 2016) before applying the same cutoff, assuming the true baseline corresponds to the region where the majority of markers are located. These input groups provide insights into diverse baseline normalization challenges, as depicted in Figure 2a.

In cases with a correctly positioned baseline and moderate CNA load, the logR-based and density-based methods are expected to produce largely concordant results, as the core assumptions of both approaches are met. However, complications arise when these assumptions are violated. The accuracy of these calls can, of course, still be affected by other factors such as data quality, but our focus here is on discrepancies arising from baseline estimation. The logR-based calling performs well with correct baselines but struggles with shifted baselines, while the density-based calling is robust to baseline shifts but fails when the assumption of marker clustering around the true baseline is invalid. For such challenging profiles, *CNAdjust* enhances calling quality by aligning results with higher posterior probabilities, producing baseline-corrected outputs. Substantial discrepancies between adjusted and original calls serve as indicators of baseline abnormalities in the original profiles. In this study, the calling state for segments spanning at least half of a chromosome arm was considered as the arm-level calling state, and samples with at least one arm-level discrepancy between original and adjusted calls were classified as having baseline issues.

#### 3.1.1 Closer alignment with absolute ploidy-corrected copy changes

Our validation, illustrated in Figure 2b, focused on these samples with baseline issues. Specifically, 447 samples failed under the logR-based approach, 2,035 failed under the density-based approach, and 202 failed under both methods. Analysis of segment value distributions revealed deviations from the expected maximum signal peak of 0 in the logR-based group, consistent with the third and fourth segment profile types depicted in Figure 2a. Similarly, the emergence of multiple major peaks in the density-based group aligns with expectations, as shown in the second and fourth profile types in Figure 2a. Additionally, other non-adjusted samples exhibited a distinct normal distribution of segment values (Supplementary Figure S2), further supporting the necessity and efficacy of *CNAdjust*. These findings underscore the unique challenges of each calling method and demonstrate the robust ability of *CNAdjust* to resolve baseline issues effectively.

Application of the workflow to the different input calling methods yielded distinct effects on the overall CNA fraction, as illustrated in the middle panel of Figure 2b. For the logR-based group, the adjustment led to a universally pronounced decrease in the CNA fraction. In contrast, the effect on the density-based group was more project-dependent. For example, while adjustments affected over 30% of calls in the TCGA-SARC and TCGA-CHOL datasets for the logR-based group, the impact on the density-based group was more variable: in the TCGA-KICH and TCGA-ACC projects, over 50% of samples experienced an increase in their CNA fraction post-adjustment, whereas in the TCGA-LUSC, TCGA-UCS, and TCGA-ESCA projects, over 30% of samples showed a decrease.

Validation against absolute copy number estimates further confirmed the effectiveness of our adjustment strategy (Figure 2b, right panel). For this comparison, we used absolute copy number and ploidy estimates generated for the same samples by the ABSOLUTE algorithm (Carter et al., 2012). To ensure consistency, these absolute estimates were summarized at the chromosome-arm level by calculating weighted averages based on segment lengths. In problematic samples, the original calling states exhibited ambiguous copy change amplitudes that lacked clear alignment with these absolute estimates. After adjustment, our workflow significantly enhanced the clarity of ploidy-corrected copy change amplitudes, achieving a much closer alignment with the absolute copy number data. In contrast, non-adjusted samples retained distinct ploidy-corrected copy change patterns across different calling states (Supplementary Figure S2), underscoring the value of the baseline correction.

To confirm that these improvements are driven by the integration of biologically accurate information, we performed a control experiment using a “least-likely prior.” This counter-evidential prior was constructed by identifying, for each genomic region, the CNA state with the lowest probability in the original cohort data and assigning it a very high probability (0.98), while the other two states were assigned low probabilities (0.01). When *CNAdjust* was run with this actively misleading prior, performance degraded catastrophically, as illustrated in Supplementary Figure S3. Instead of reducing the overall CNA fraction, the adjustment process now significantly increased it across both input methods. Furthermore, the alignment with absolute copy number estimates was largely lost. This result validates that the success of *CNAdjust* relies on the correct interplay between the cohort-specific prior and the study-specific data, and is not an artifact of the adjustment framework itself.

#### 3.1.2 Enhanced CNA pattern interpretation

**FIGURE 3:**
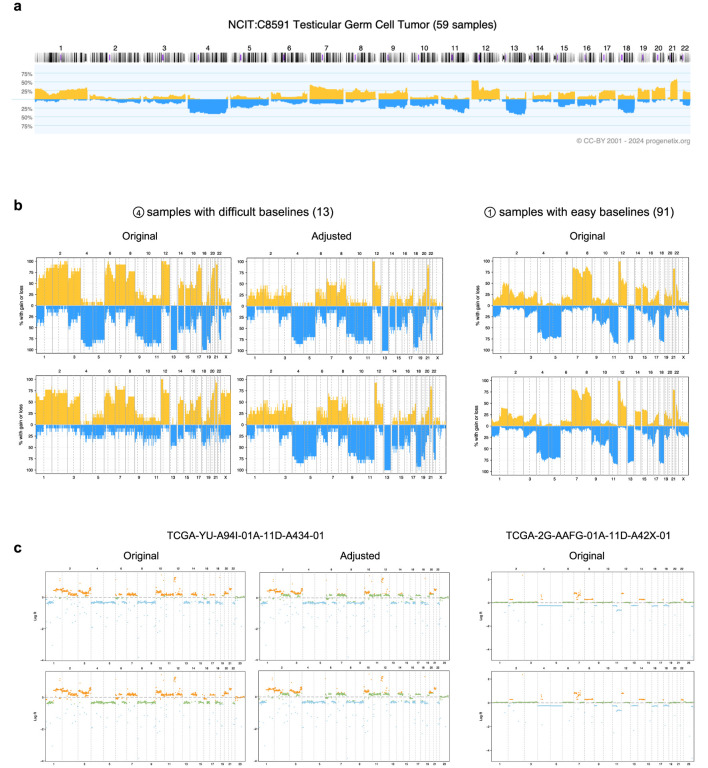
Effects of adjustment in the TCGA-TGCT cohort. **(a)** The prior CNA frequency plot for testicular germ cell tumors, sourced from the Progenetix database. **(b)** CNA frequency plots from TCGA-TGCT samples, categorized by input calling method (rows) and baseline quality (columns). The top and bottom rows correspond to the logR-based and density-based methods, respectively. The samples are grouped into 13 with “difficult baselines” (left, corresponding to profile type 4 in Figure 2a) and 91 with “easy baselines” (right, profile type 1). **(c)** Individual segment profiles for representative samples from the groups in panel **(b)**. The left column shows a difficult sample before and after adjustment, illustrating the correction of baseline errors. The right column shows an easy sample for comparison. CNA states are indicated by color: blue for deletions, orange for duplications, and green for copy-neutral states.

To illustrate the improved interpretation achieved through baseline adjustment, we examined data from the TCGA testicular germ cell tumor (TCGA-TGCT) project specifically. Among the 155 samples analyzed, 91 displayed consistent CNA callings across both input groups after adjustment, as these samples did not exhibit baseline issues. Despite originating from distinct calling strategies, these samples showed highly similar CNA patterns (Figure 3b). Notably, key CNA features, such as deletions on chromosomes 4, 5, 11, 13q, and 18, along with duplications on chromosomes 7, 8, 12p, and 21q, were readily identifiable. These features aligned closely with prior CNA patterns from Progenetix used for adjustment (Figure 3a).

In contrast, 13 samples with baseline issues posed calling challenges, as both methods initially failed to produce reliable results. Their original CNA patterns deviated substantially from those of consistent samples, with the two calling methods yielding conflicting outputs: the logR-based input tended to overestimate total CNAs, while the density-based input introduced a duplication bias. These discrepancies obscured key characteristic patterns, complicating biological interpretation.

Following adjustment, our workflow resolved these inconsistencies by integrating prior cohort-level CNA patterns with study-specific logR distributions (Figures 3b,c). The adjusted CNA patterns aligned more closely with expected biological characteristics, effectively revealing features such as the hallmark 12p duplication—a genetic signature of testicular germ cell tumors (Batool et al., 2019)—that had been previously obscured. These results underscore the capability of *CNAdjust* in handling complex cases and provide more accurate and biologically meaningful CNA interpretations.

### 3.2 Aneuploidy’s impact on baseline ambiguity

**FIGURE 4:**
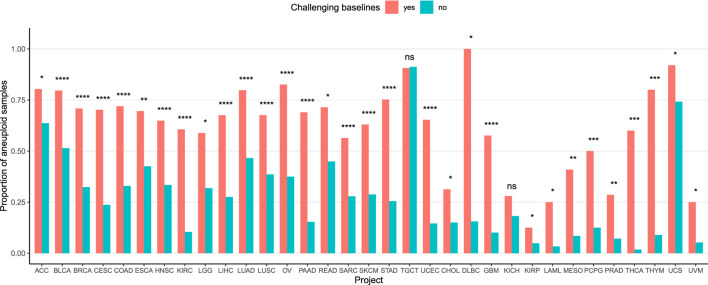
Association between aneuploidy and baseline complexity. The Y-axis represents the proportion of aneuploid samples in each group. Samples with arm-level calling differences post-adjustment, regardless of the calling strategy, were categorized as having baseline issues (red), while other samples were considered baseline-stable (blue). *P*-values were calculated using a Chi-squared test, except for the 13 rightmost projects where Fisher’s exact test was used due to low event counts.

To investigate the influence of aneuploidy on baseline normalization challenges, we analyzed its association with the baseline abnormalities identified by our method. Samples with ploidy ≥ 2.5 or ≤ 1.5 were classified as aneuploid. We then tested for a statistical association between aneuploidy and baseline ambiguity within each TCGA project. As summarized in Figure 4, we observed that samples with challenging baselines (red bars) consistently had a higher proportion of aneuploidy than those with stable baselines (blue bars). This association between aneuploidy and baseline ambiguity was statistically significant in 31 of the 33 projects. These findings underscore the critical impact of aneuploidy on baseline accuracy and highlight the need for adjustment methods, like *CNAdjust*, that can address complex karyotypic variations.

## 4 Discussion

*CNAdjust* provides an automated and systematic solution to address baseline inaccuracies in CNA calling, a prevalent and often overlooked challenge in cancer genomics. This method is designed to enhance calling accuracy and effectively handle the complexities of large, heterogeneous datasets. Its robustness was demonstrated through its application to the TCGA pan-cancer dataset, where we analyzed around 10,000 samples across 33 tumor types derived from divergent calling methodologies. This strategy highlighted various instances of ambiguous baselines and validated the fundamental logic of our approach: samples flagged for baseline issues exhibited abnormal logR distributions, and our adjustments led to improved alignment with absolute copy number estimates. A focused analysis of the TCGA-TGCT cohort further underscored CNAdjust’s utility, showing how it can restore characteristic CNA patterns obscured by baseline errors. Finally, the strong association we found between aneuploidy and baseline ambiguity across nearly all TCGA projects emphasizes that addressing karyotypic complexity is critical for accurate cancer CNA studies.

Despite its strengths, *CNAdjust* has limitations. A key assumption is that segment values for a given CNA state are drawn from a common distribution within a study. This may not hold if a study contains highly aberrant samples that do not appear as statistical outliers, which could complicate the adjustment. To mitigate this, our method incorporates a reference logR distribution when more than 50% of a study’s samples are flagged as abnormal and adjusts the fitted distributions when this fraction exceeds 25% (detailed in Supplementary Section 3).

Another consideration is the reliance on appropriate prior selection, which raises a valid concern about intra-cohort heterogeneity. It is true that applying a single, genome-wide prior to a diverse cancer cohort is a simplification, as molecular subtypes with distinct CNA patterns exist (Yang et al., 2024). However, it is crucial to emphasize that the prior in our Bayesian framework does not rigidly force profiles to conform to a cohort average. Instead, it acts as a probabilistic guide that is balanced against the data-driven plausibility score from the sample itself. For this pan-cancer study, we employed broad NCIt-based cohort definitions with frequency data from Progenetix (accessed via the *pgxRpi* (Zhao and Baudis, 2025) package) as a pragmatic and scalable approach to demonstrate the method’s utility. Recognizing that the relationship between CNA heterogeneity and tumor classification is not always straightforward, the *CNAdjust* framework was designed for flexibility. Its performance can be potentially enhanced by incorporating more granular priors, such as those defined by known molecular subtypes or other user-defined biological knowledge. This adaptability is a core strength, allowing for a more tailored and precise adjustment process as cohort definitions become more refined.

## 5 Conclusion

*CNAdjust* effectively improves the accuracy of relative CNA calls by systematically identifying and correcting baseline inaccuracies. This study underscores that proper baseline determination is critical for the accurate interpretation of CNA patterns, which in turn is essential for advancing our understanding of tumor biology and informing clinical applications. In the era of big data, the demand for scalable, efficient, and automated solutions like *CNAdjust* is paramount. By enhancing the quality and reliability of CNA analyses, our method represents a significant advancement towards the construction of harmonized reference datasets and provides a valuable tool to support the progress of large-scale genomic research and precision medicine.

## Data Availability

The datasets analyzed in this study are publicly available. The TCGA pan-cancer datasets used for validation were downloaded from the GDC Data Portal https://portal.gdc.cancer.gov/). The data used for method development, including the GEO series for parameter optimization and the cohort-specific CNA frequency data, were sourced from the Progenetix oncogenomic resource (https://progenetix.org). Further details on the specific TCGA projects and GEO series used are provided in the Supplementary Material.

## Footnotes

1 Alternatively the term Copy Number Variations (CNV) can be employed, potentially with added “somatic” specifier to disambiguate from inherited variants.
