# Supplementary material for: CNAdjust: enhancing CNA calling accuracy through systematic baseline adjustment
Source: Front Genet. 2025 Sep 26;16:1674138. doi: 10.3389/fgene.2025.1674138 (PMC12510678; doi:10.3389/fgene.2025.1674138)
Supplement: Supplementary file 1 [file DataSheet1.pdf]

Supplementary file

CNAdjust: Enhancing CNA Calling Accuracy through Systematic Baseline Adjustment

Hangjia Zhao[1,2*] and Michael Baudis[1,2*]

[1] Department of Molecular Life Sciences, University of Zurich, Winterthurerstrasse 190, 8057, Zurich, Switzerland and [2] Computational Oncogenomics Group, Swiss Institute of Bioinformatics, Winterthurerstrasse 190, 8057, Zurich, Switzerland
[*]Corresponding author

Section 1 Default Parameter Determination in Test Series

Section 1.1 Test Series Collection

The test series comprises 36 series originally sourced from the Gene Expression Omnibus (GEO) database [1]. Corresponding segment data were accessed from the arrayMap cohort in the Progenetix resource [2]. These series are highly heterogeneous, covering various tumor types and measurement platforms. Detailed information about these series can be found in Supplementary Table S1.

Section 1.2 Parameter Determination

Three parameters are essential for identifying potentially problematic samples: weighted standard deviation of logR values by associated marker number, CNA fraction, and the ratio between duplication and deletion fractions. We analyzed the distribution of these values across the test series, with results presented in Supplementary Figures S1. It is important to note that this workflow aims to identify samples suspected of having issues, rather than definitively problematic ones. Therefore, default parameters are set to select outliers exhibiting large CNA fractions, high weighted SD of logR, and skewed fraction ratios. Specifically, samples with weighted SD > 0.35, CNA fraction > 0.5, and fraction ratio > 3 or < 1/3 are identified as potentially abnormal.

Additionally, we observed that different calling approaches generate varying CNA fractions and fraction ratios. LogR-based calling tends to overcall, resulting in higher CNA fractions, while density-based calling is more likely to exhibit a bias towards either duplication or deletion. To enhance sensitivity, two additional criteria have been established: a CNA fraction > 0.7 to identify overcalled samples and a CNA fraction > 0.2 with a fraction ratio > 5 or < 1/5 to identify samples with moderate CNA calls but extremely skewed fraction ratios.

For profiles with more than 1000 segments, baseline adjustment is generally not considered due to the likelihood of high noise being a more significant issue. As a workaround, the workflow applies a cutoff using various combinations of thresholds (0.1, 0.15, 0.3 for low-level calling; 1, 1.5, 2 for high-level calling) to attempt better call accuracy. All these numeric parameters can be tuned in the *nextflow.config* file based on users' data distribution or prior knowledge.

Section 2 TCGA Data Collection

Masked copy number segment profiles were downloaded using the *TCGAbiolinks* R package [3]. The absolute allele-specific copy number profiles and ploidy estimates were obtained from the GDC PanCanAtlas Publication page (https://gdc.cancer.gov/about-data/publications/pancanatlas). The project names, sample numbers, and corresponding National Cancer Institute Thesaurus (NCIt) terms [4] are detailed in Supplementary Table S2. The mapping of NCIt terms is primarily based on tumor names. For projects associated with multiple NCIt terms, we assessed the CNA frequency patterns in Progenetix for each term. Given that these patterns were found to be similar, the term with the highest number of corresponding samples in Progenetix was selected.

Section 3 Segment Value Distribution Modification

When problematic samples are not statistical outliers within the same study, the fitted segment value distribution may lose its utility in guiding adjustments effectively. To address this, we implement specific methodologies for modifying segment value distributions under defined conditions:

1. Reference Distribution Implementation: If over 50% of the samples are identified as potentially baseline problematic or are over-segmented (exceeding 1000 segments by default), a reference distribution replaces the fitted distribution for adjustments. This reference is derived from around 26,000 heterogeneous tumor sample segment profiles from Progenetix that are not identified as problematic.

2. Data Refitting: When more than 25% of the samples are considered potentially baseline problematic, the data undergoes refitting. This involves adjusting all problematic samples with a bias in one direction (e.g., duplication bias) while maintaining the original state of samples with a different bias (e.g., deletion bias). We then calculate the posterior probability of the entirely shifted data and compare it to the posterior probability of the original data to decide if a total shift should be applied. If beneficial, the data value distribution is replaced by this refitted distribution.

Section 4 Supplementary Figures

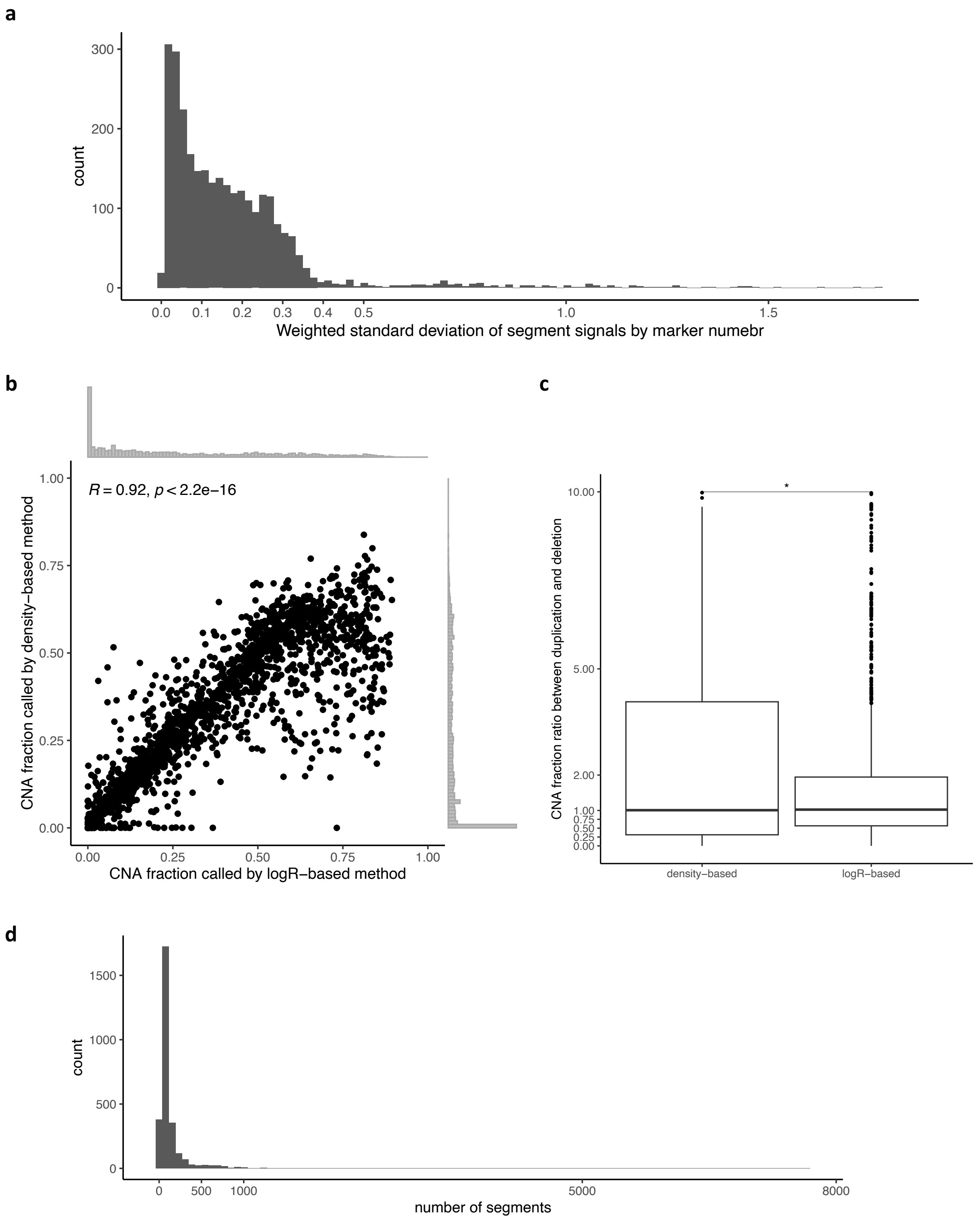

Supplementary Figure S1: Overview of segment data from test series. a Distribution of weighted standard deviation of logR values by marker number across all samples. **b** Scatter plot with marginal histograms showing CNA fraction generated by different calling methods. Each dot represents one sample. **c** Fraction ratio between duplication and deletion. **d** Distribution of segment number per sample.

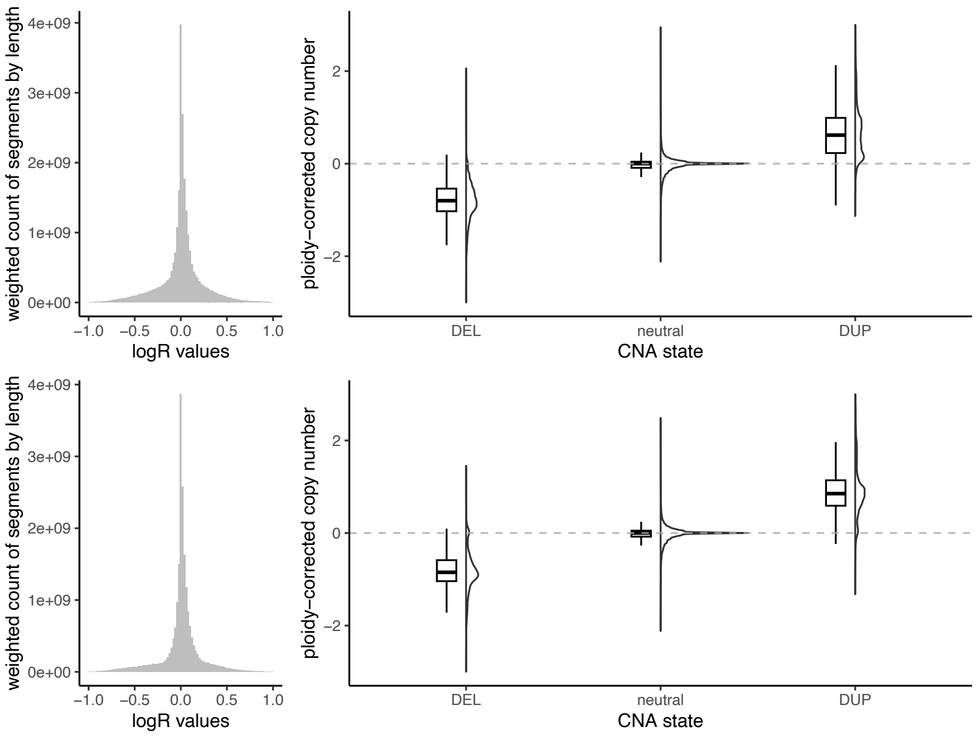

Supplementary Figure S2: Overview of TCGA samples without baseline adjustment. The top panel shows results from the logR-based input group. The bottom panel shows results from the density-based input group.

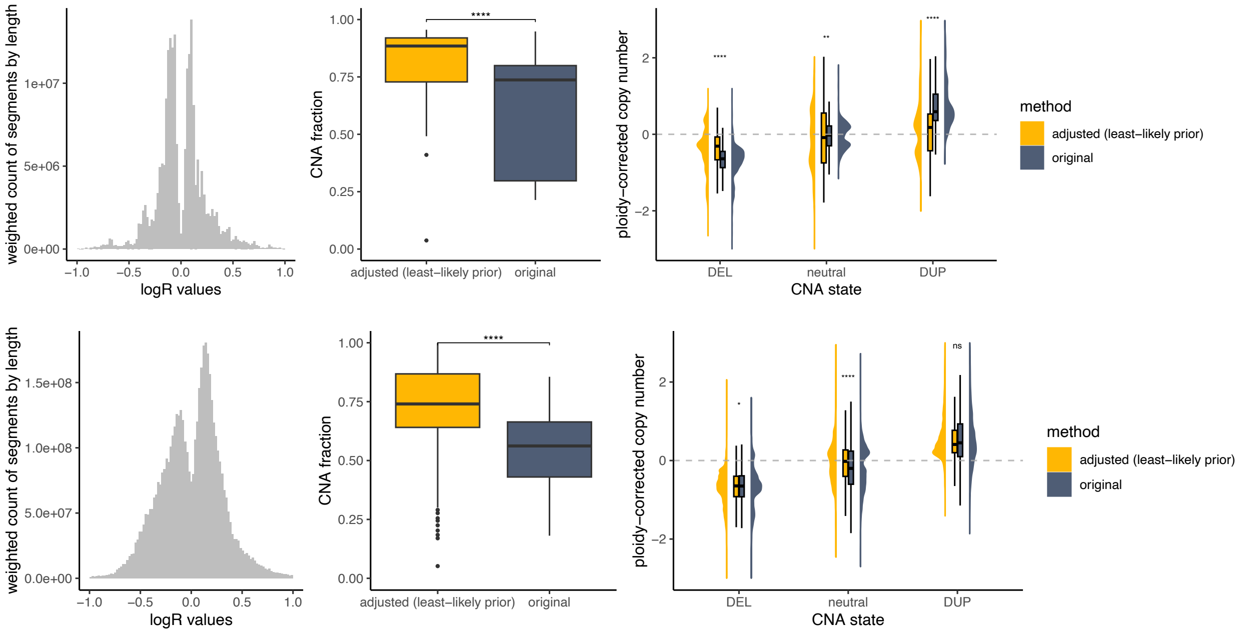

Supplementary Figure S3: Validation through a "least-likely prior" control experiment. The top and bottom panels correspond to samples with baseline issues from the logR-based and density-based calling groups, respectively. The left plot shows weighted histograms of logR values (weighted by segment length). The middle plot illustrates CNA fractions; statistical significance was determined using a paired t-test with Benjamini-Hochberg correction. The right plot depicts ploidy-corrected copy changes across different calling states; statistical significance was determined using a t-test with Benjamini-Hochberg correction.

Section 5 Supplementary Tables

Supplementary Table S1: Overview of test GEO samples

Series	Sample number	Disease type	Platform
GSE10099	626	Ductal Breast Carcinoma	GPL2004,GPL2005
GSE11782	10	Oropharyngeal Squamous Cell Carcinoma, Unspecified Tissue	GPL2641
GSE12019	225	Ductal Breast Carcinoma, Lung Non-Small Cell Carcinoma Unspecified Tissue	GPL3720
GSE13429	49	Unspecified Tissue	GPL3718,GPL6801
GSE16121	45	Plasmacytoma	GPL2005
GSE16668	7	Ductal Breast Carcinoma	GPL8720
GSE16746	100	Chronic Lymphocytic Leukemia	GPL3718
GSE17534	104	Cutaneous Melanoma, Unspecified Tissue	GPL3720
GSE19539	72	Ovarian adenocarcinoma	GPL6801
GSE19996	48	B Lymphoblastic Leukemia/Lymphoma with t(12;21)(p13.2;q22.1); ETV6-RUNX1, B Acute Lymphoblastic Leukemia	GPL3718,GPL3720
GSE20353	86	Acute Lymphoblastic Leukemia	GPL3718
GSE21349	78	Plasmacytoma, Unspecified Tissue	GPL3718,GPL3720
GSE22305	22	Cutaneous Melanoma, Amelanotic Melanoma, Unspecified Tissue	GPL6801
GSE22615	15	Pituitary Gland Carcinoma, Unspecified Tissue	GPL6801
GSE22789	67	Gastric Adenocarcinoma	GPL7394
GSE26674	6	Chondrosarcoma, Chondroma	GPL6801
GSE30283	56	Ovarian Serous Cystadenocarcinoma	GPL6801
GSE30754	4	Bladder Urothelial Carcinoma	GPL5477
GSE31272	34	Plasmacytoma	GPL6801
GSE32058	62	Squamous Cell Lung Carcinoma	GPL2879
GSE35189	121	Ductal Breast Carcinoma	GPL9128
GSE39130	65	Ovarian Serous Cystadenocarcinoma, Unspecified Tissue	GPL3718
GSE39380	17	Plasma Cell Leukemia	GPL3718
GSE41646	116	Acute Myeloid Leukemia Not Otherwise Specified, Unspecified Tissue	GPL6801
GSE43060	64	Acute Lymphoblastic Leukemia	GPL3718,GPL3720
GSE43933	44	Colon Adenocarcinoma, Unspecified Tissue	GPL16573
GSE45573	37	Leiomyosarcoma, Soft Tissue Sarcoma	GPL8841
GSE50667	9	Bladder Urothelial Carcinoma, Unspecified Tissue	GPL6801
GSE63236	50	Unspecified Tissue	GPL3718
GSE68999	68	Plasma Cell Myeloma, Unspecified Tissue	GPL6801

GSE7068	23	Lung Small Cell Carcinoma	GPL2004,GPL2005
GSE7882	50	Ductal Breast Carcinoma In Situ,<br>Lobular Breast Carcinoma In Situ	GPL5326
GSE80314	5	Acute Myeloid Leukemia Not Otherwise Specified,<br>Therapy-Related Myeloid Neoplasm	GPL18637
GSE93886	23	Acute Myeloid Leukemia Not Otherwise Specified	GPL16131
GSE9585	68	Skin Squamous Cell Carcinoma,<br>Ductal Breast Carcinoma,<br>Colon Adenocarcinoma,<br>Gastric Adenocarcinoma,<br>Cutaneous Melanoma,<br>Ovarian Carcinoma,<br>Pharyngeal Squamous Cell Carcinoma,<br>Renal Cell Carcinoma,<br>Lung Carcinoma,<br>Pancreatic Adenocarcinoma,<br>Prostate Adenocarcinoma	GPL2005,GPL2004,GP<br>L3718,GPL3720
GSE9635	374	Glioblastoma,<br>Anaplastic Oligodendroglioma,<br>Anaplastic Astrocytoma,<br>Mixed Glioma,<br>Unspecified Tissue	GPL2004,GPL2005

Supplementary Table S2: Overview of TCGA samples

Project Abbreviation	Project name	Sample number	NCIt code
ACC	Adrenocortical carcinoma	89	NCIT:C9325
BLCA	Bladder Urothelial Carcinoma	402	NCIT:C39851
BRCA	Breast invasive carcinoma	1030	NCIT:C9245
CESC	Cervical squamous cell carcinoma and endocervical adenocarcinoma	296	NCIT:C4028
CHOL	Cholangiocarcinoma	36	NCIT:C4436
COAD	Colon adenocarcinoma	394	NCIT:C4349
DLBC	Lymphoid Neoplasm Diffuse Large B-cell Lymphoma	47	NCIT:C8851
ESCA	Esophageal carcinoma	163	NCIT:C3513
GBM	Glioblastoma multiforme	380	NCIT:C3058
HNSC	Head and Neck squamous cell carcinoma	495	NCIT:C34447
KICH	Kidney Chromophobe	61	NCIT:C4146
KIRC	Kidney renal clear cell carcinoma	330	NCIT:C4033
KIRP	Kidney renal papillary cell carcinoma	270	NCIT:C6975
LAML	Acute Myeloid Leukemia	124	NCIT:C3171
LGG	Brain Lower Grade Glioma	510	NCIT:C3059
LIHC	Liver hepatocellular carcinoma	361	NCIT:C3099
LUAD	Lung adenocarcinoma	495	NCIT:C3512
LUSC	Lung squamous cell carcinoma	468	NCIT:C3493
MESO	Mesothelioma	81	NCIT:C3786
OV	Ovarian serous cystadenocarcinoma	397	NCIT:C7978
PAAD	Pancreatic adenocarcinoma	166	NCIT:C8294

PCPG	Pheochromocytoma and Paraganglioma	164	NCIT:C3326
PRAD	Prostate adenocarcinoma	470	NCIT:C2919
READ	Rectum adenocarcinoma	139	NCIT:C9383
SARC	Sarcoma	248	NCIT:C9118
SKCM	Skin Cutaneous Melanoma	460	NCIT:C3510
STAD	Stomach adenocarcinoma	423	NCIT:C4004
TGCT	Testicular Germ Cell Tumors	155	NCIT:C8591
THCA	Thyroid carcinoma	456	NCIT:C4815
THYM	Thymoma	106	NCIT:C3411
UCEC	Uterine Corpus Endometrial Carcinoma	494	NCIT:C40179
UCS	Uterine Carcinosarcoma	56	NCIT:C42700
UVM	Uveal Melanoma	80	NCIT:C7712

Reference

[1] Barrett, T., Wilhite, S.E., Ledoux, P., Evangelista, C., Kim, I.F., Tomashevsky, M., Marshall, K.A., Phillippy, K.H., Sherman, P.M., Holko, M., *et al.*: Ncbi geo:archive for functional genomics data sets—update. Nucleic acids research **41**(D1), 991–995 (2012)

[2] Huang, Q., Carrio-Cordo, P., Gao, B., Paloots, R., Baudis, M.: The progenetix oncogenomic resource in 2021. Database **2021**, 043 (2021)

[3] Colaprico, A., Silva, T.C., Olsen, C., Garofano, L., Cava, C., Garolini, D., Sabedot, T.S., Malta, T.M., Pagnotta, S.M., Castiglioni, I., *et al.*: Tcgabiolinks: an r/bioconductor package for integrative analysis of tcga data. Nucleic acids research **44**(8), 71–71 (2016)

[4] National Cancer Institute: NCI Thesaurus (2024). https://ncithesaurus.nci.nih.gov/
